# Mechanical power and VILI: navigating heterogeneity in critical illness

**DOI:** 10.1186/s40635-025-00760-w

**Published:** 2025-05-15

**Authors:** Glauco M. Plens, Eduardo Leite Vieira Costa

**Affiliations:** 1 Laboratório de Pneumologia LIM-09, Divisao de Pneumologia, Faculdade de Medicina, Instituto Do Coracao, Hospital das Clinicas HCFMUSP, Universidade de Sao Paulo, Av. Dr. Arnaldo, 455, Sala 2144 (2nd Floor), 01246-903 Sao Paulo, Brasil; 2 Department of Medicine, Division of Respirology, University Health Network, Toronto, Canada; 3 Research and Education Institute, Hospital Sírio-Libanês, Sao Paulo, Brazil

Several ventilatory variables have been studied as markers of lung stress and strain. Tidal volume, which was initially thought to be the main factor responsible for ventilator-induced lung injury (VILI), has been replaced by driving pressure and mechanical power in the last decade [1, 2]. In addition, a simple parameter combining driving pressure and respiratory rate (4∆*P* + RR) has shown equivalent accuracy compared to mechanical power to predict mortality in patients with acute respiratory distress syndrome (ARDS) [3]. However, most studies reporting an association between ventilatory variables and clinical outcomes rely on measurements from the first 24 h after randomization, and little is known about how time can influence this association. Moreover, the association of these variables with mortality in other pathologies remains uncertain. 

In this issue of the journal, Wu and colleagues report a significant impact of mechanical power on oxygenation and ventilator-free days at day 28 in a heterogeneous cohort of clinical and surgical patients [4]. Their work highlights how measures of lung stress and strain could be relevant in various diseases, with mechanical power being associated with outcomes even in disease states where acute respiratory failure might not be the primary reason for intubation. Furthermore, they report a time-dependent aspect of the safe threshold of mechanical power. By looking into different cohorts based on primary diagnosis, the authors make a case for different magnitudes of association between mechanical power and clinical outcomes. These findings support the rationale of precision ventilation with individualized targets according to different diseases and disease progression. However, despite the thoughtful causal diagram provided by the authors, these associations are still susceptible to residual confounding due to study design limitations and should be confirmed in future prospective studies.

Notably, Wu and colleagues used the PaO_2_/FiO_2_ ratio as a mediator between mechanical power and ventilator-free days. However, the relationship among these variables may be more complex than anticipated. Oxygenation can delay extubation not only due to underlying severity of illness, but also because clinicians frequently use PaO_2_/FiO_2_ thresholds to guide decisions about ventilator. Furthermore, while PaO_2_/FiO_2_ reflects the degree of gas exchange impairment, it is not necessarily causally related to VILI. In fact, injurious ventilation characterized by high lung stress and strain can initially improve oxygenation as demonstrated in the ARMA trial [5]. VILI seems to be more related to ventilation intensity variables, as driving pressure and respiratory rate [3].

This study underscores the importance of designing trials that account for the individualized physiology of heterogeneous patient populations and disease states. While conducting prospective trials to address such complexity might be a formidable challenge, clinicians can also rely on physiological principles and clinical experience to guide bedside decision-making when high-quality evidence is lacking.

## Data Availability

Not applicable.
